# The infected fracture: can we agree on standard definitions?

**DOI:** 10.1097/OI9.0000000000000057

**Published:** 2020-03-23

**Authors:** David J. Hak

**Affiliations:** Hughston Orthopedic Trauma Surgeons, Central Florida Regional Hospital, Sanford, FL. University of Central Florida, Orlando, FL.

**Keywords:** infection after fracture fixation, infection consensus definition, infection standard definition

## Abstract

A precise definition of infection after fracture fixation is essential for the evaluation of published research data and for the future establishment of uniform treatment concepts. Recently, a multidisciplinary expert panel has developed a consensus definition that includes 4 confirmatory criteria for infection following fracture fixation. These criteria are: Fistula, sinus, or wound breakdown; purulent drainage or deep purulence at surgery; positive cultures from at least 2 separate deep tissue/implant specimens taken during an operative intervention; and microorganisms in deep tissue specimens confirmed by histopathological staining. The consensus panel also identified 6 categories of suggestive criteria which are features associated with infection that requires further investigation.

Infection following fracture fixation is a challenging problem that has significant impacts on patients and healthcare costs.^[1]^ Infection following fracture fixation commonly requires one or more repeat operations, prolonged treatment with antibiotics, and a prolonged and uncertain time to full recovery. Eradication of infection may not always be possible, leading to prolonged treatment involving multiple operative procedures, loss of function, and even amputation. It is important to have a precise definition of infection after fracture fixation for the evaluation of published research data and for the future establishment of uniform treatment concepts.

While a consensus definition for periprosthetic joint infection had been developed in 2014, no such consensus definition existed for infection following fracture fixation until 2018.^[2,3]^ In an international survey of trauma surgeons, 66% agreed that prosthetic joint infection and fracture-related infection are not equal with respect to diagnosis, treatment, and outcome.^[4]^ In this survey, 90% of respondents agreed that a consensus-derived definition for fracture-related infection is required. In regards to the specific criteria that should be included in the definition, 75% of respondents indicated it should include a positive culture, 65% indicated that there should be an elevated C reactive protein, 61% indicated that there should be purulent drainage, and 57% indicated that should be local signs of infection.

Evaluation of inflammatory markers is commonly performed in cases of suspected infection following fracture fixation; however following trauma these can be difficult to interpret since their elevation is not specific to infection. Investigators reviewed 168 patients who presented with a suspected fracture-related infection at 2 level I trauma centers examining the diagnostic accuracy of individual serum inflammatory markers (C-reactive protein, white blood cell count, and erythrocyte sedimentation rate) in defining infection.^[5]^ Fracture-related infection was defined as a positive microbiology result from a surgically obtained tissue sample, and absence of infection was defined as absence of clinical infection symptoms at follow-up of at least 6 months. The investigators found that C-reactive protein value had 38% sensitivity, 34% specificity, 42% positive predictive value (PPV), 78% negative predictive value (NPV), and a diagnostic accuracy of 52%. White blood cell count had 39% sensitivity, 74% specificity, 46% PPV, 67% NPV, and diagnostic accuracy of 61%. Erythrocyte sedimentation rate had 62% sensitivity, 64% specificity, 45% PPV, 76% NPV, and diagnostic accuracy of 80%. The diagnostic value of erythrocyte sedimentation rate in the current study appears to be high, with an accuracy of 80%; however, there was large overlap in the interquartile range in patients with and without infection indicating the discriminative value to be low. The investigators concluded that the added diagnostic value of C-reactive protein, white cell count, and erythrocyte sedimentation rate was limited. They noted that infection following fracture fixation could still be present when serum inflammatory markers are within normal range and advocated interpreting the results with caution in patients with a suspected infection following fracture fixation.

A systematic review of the scientific literature was performed to identify the definitions used to describe infectious complications after internal fixation of fractures. The investigators identified 100 published fracture fixation randomized controlled trials and collected data on the studies’ definition of infectious complications after fracture fixation. Only 2 of the studies cited a validated definition to describe fracture related infection, while in 28 trials authors used a self-designed definition. In the other 70 studies the publication reported no description of an infection definition, although all described infection as an outcome parameter.^[6]^

Recently, a panel of multidisciplinary experts have developed a consensus definition for infection after fracture fixation.^[3]^ The consensus process consisted of 3 phases. In the first phase experts exchanged ideas using a modified Delphi process through videoconferences and email.^[7]^ During this phase 4 main topics (classification, location, terminology, and diagnostic criteria) were identified as critical in providing knowledge and standards for the definition.

In the second phase, participants were brought together for a face-to-face meeting in December 2016 to address the specific topics that were agreed upon in the first phase and to vote on resolutions. Participants included representatives from various international organizations (AO Foundation, European Bone, and Joint Infection Society) and prominent orthopaedic trauma hospitals and academic centers that have a major interest in fracture-related infections. These experts were multidisciplinary specialists including infectious disease physicians, orthopaedic trauma surgeons, and clinical pathologists. During the meeting, separate sessions addressed each of the 4 main topics of classification, location, terminology, and diagnostic criteria. A consensus definition of infection after fracture fixation was proposed and further clarified in the third and final phase that consisted of completion of the publication.

The multidisciplinary panel defined 2 levels of certainty around diagnostic features for infection after fracture fixation. These were confirmatory criteria that identified an infection was definitely present, and suggestive criteria which are features associated with infection that require further investigation.

The 4 confirmatory criteria of this consensus definition are:

1. Fistula, sinus, or wound breakdown (with communication to the bone or the implant).
2. Purulent drainage from the wound or presence of pus during surgery.
3. Phenotypically indistinguishable pathogens identified by culture from at least 2 separate deep tissue/implant (including sonication-fluid) specimens taken during an operative intervention. In case of tissue, multiple specimens (≥3) should be taken, each with clean instruments (not superficial or sinus tract swabs). In cases of joint effusion, arising in a joint adjacent to a fractured bone, fluid samples obtained by sterile puncture may be included as a single sample.
4. Presence of microorganisms in deep tissue taken during an operative intervention, as confirmed by histopathological examination using specific staining techniques for bacteria or fungi.^[3]^

The suggestive criteria that the consensus panel identified which are features associated with infection that requires further investigation were described in 6 categories:

1. Clinical signs: pain without weight bearing, or increasing over time, or new-onset; local redness; local swelling; increased local temperature; and fever ≥ 38.3 C.
2. Radiological signs: bone lysis at the fracture site or around the implant, implant loosening, sequestration occurring over time, failure of progression of bone healing, periosteal bone formation at localizations other than the fracture site, or in cases of a consolidated fracture.
3. A pathogenic organism identified by culture from a single deep tissue/implant specimen taken during an operative intervention.
4. Elevated serum inflammatory markers (a secondary rise after an initial decrease following trauma or a consistent elevation over a period in time): erythrocyte sedimentation rate, white blood cell count, C-reactive protein.
5. Persistent, increasing, or new onset wound drainage, beyond the first few postoperative days.
6. New-onset joint effusion in patients with an intra-articular fracture or when an implant penetrates the joint capsule.

The work of the expert panel that developed a consensus definition has built a path forward in defining infection after fracture fixation. Future clinical studies using this consensus definition will be needed to validate its usefulness and to further define the relevance of suggestive criteria.
